# Evaluation of Droplet Digital PCR Assay for the Detection of Microsatellite Instability in Colorectal, Gastric, and Endometrial Cancers

**DOI:** 10.3390/diagnostics16101550

**Published:** 2026-05-20

**Authors:** Yousun Chung, Sujin Oh, Soo Kyung Nam, Hyunji Kim, Cheol Lee, Gyeong Hoon Kang, Hyeon Jeong Oh, Hye Seung Lee, Kyoung Un Park

**Affiliations:** 1 Department of Laboratory Medicine, Kangdong Sacred Heart Hospital, Seoul 05355, Republic of Korea; yousun623@gmail.com; 2 Department of Laboratory Medicine, Seoul National University College of Medicine, Seoul 03080, Republic of Korea; tnwls5976@snu.ac.kr; 3 Department of Interdisciplinary Program in Cancer Biology, Seoul National University College of Medicine, Seoul 03080, Republic of Korea; 4 Cancer Research Institute, Seoul National University College of Medicine, Seoul 03080, Republic of Korea; 5 Department of Laboratory Medicine, Seoul National University Bundang Hospital, Seongnam 13620, Republic of Korea; 6 Department of Pathology, Seoul National University Hospital, Seoul National University College of Medicine, Seoul 03080, Republic of Korea; fe98134@snu.ac.kr; 7 Department of Pathology, Seoul National University Bundang Hospital, Seoul National University College of Medicine, Seongnam 13620, Republic of Korea

**Keywords:** microsatellite instability, droplet digital PCR, colorectal cancer, gastric cancer, endometrial cancer

## Abstract

**Background**: Microsatellite instability (MSI) is an important biomarker for the diagnosis of Lynch syndrome and for guiding immunotherapy in various solid tumors. Droplet digital PCR (ddPCR) has emerged as a highly sensitive method for detecting MSI, particularly in circulating tumor DNA (ctDNA). This study aimed to evaluate the analytical and clinical performance of a ddPCR assay using three MSI markers (*BAT-26*, *ACVR2A*, and *DEFB105A/B*) in colorectal, gastric, and endometrial cancers. **Methods**: Formalin-fixed paraffin-embedded (FFPE) samples from 190 patients (83 colorectal, 44 gastric, and 63 endometrial cancers) and 21 plasma samples from patients with metastatic solid tumors were analyzed. MSI status determined by ddPCR was compared with conventional PCR using a pentaplex panel and immunohistochemistry (IHC). Analytical performance, including limit of blank (LoB) and limit of detection (LoD), was evaluated using cell line DNA, and clinical cut-offs were established using receiver operating characteristic analysis. **Results**: The ddPCR assay demonstrated high analytical sensitivity, with LoD values of 0.075% for *BAT-26*, 0.1% for *ACVR2A*, and 0.025% for *DEFB105A/B*. Using optimized clinical cut-offs, the concordance rate between ddPCR and conventional PCR assays was 98.4% in tissue samples. Marker performance varied by cancer type, with reduced sensitivity observed in endometrial cancer. In plasma samples, MSI-H was detected in 1 of 21 cases (4.8%), and the overall concordance rate with tissue-based MSI status was 94.7%. **Conclusions**: The ddPCR assay demonstrated high concordance with conventional MSI testing methods and showed potential as a sensitive tool for MSI detection in both tissue and plasma samples. However, optimization of marker panels and establishment of sample-type-specific clinical cut-offs are required, particularly for ctDNA-based analysis. Further large-scale studies are needed to validate the clinical utility of ddPCR for MSI detection and monitoring.

## 1. Introduction

Microsatellites are challenging to replicate accurately due to polymerase slippage on repeating units, leading to changes in allele length from insertions or deletions [1]. Normally, mismatch repair (MMR) proteins identify and correct these replication errors. However, in tumors with MMR deficiency (dMMR), these errors remain uncorrected, causing microsatellite instability (MSI) [2,3,4].

dMMR often results from hypermethylation and epigenetic silencing of the *MLH1* gene in sporadic tumors [5] or from germline mutations in one of the MMR genes in Lynch syndrome cases [6]. This deficiency is found in approximately 15–20% of colorectal cancers and several other types, including gastric, endometrial, and pancreatic cancers [6,7,8,9,10].

To screen for Lynch syndrome and to guide therapeutic decision-making, including eligibility for immune checkpoint inhibitor therapy, dMMR testing is employed in patients with solid tumors [11,12,13]. In 2017, the U.S. Food and Drug Administration (FDA) granted the first site-agnostic accelerated approval of pembrolizumab, a PD-1 inhibitor, for adult and pediatric patients with unresectable or metastatic dMMR/MSI-H solid tumors that had progressed following prior therapy, regardless of PD-L1 expression, tissue type, or tumor site [14]. This indication was subsequently converted to full approval in 2023 [15]. Given its diagnostic and predictive significance, accurate identification of dMMR status is essential for optimizing patient management and improving clinical outcomes.

The most widely used conventional methods for the detection of the status of MMR are immunohistochemistry (IHC) and PCR amplification followed by fragment analysis [16]. Recently, noninvasive diagnostic methods capable of detecting MSI in circulating tumor DNA (ctDNA) have gained considerable attention with the current trend toward systematic detection of MSI in patients with metastatic cancer. This approach enables the gaining of valuable molecular information in cases where tissue biopsy is not feasible, no measurable disease is detectable on imaging, or tumor heterogeneity compromises the representativeness of biopsy specimens. Conventional PCR methods lack the sensitivity needed to detect mutations from liquid biopsy due to the often low levels of ctDNA in plasma. Droplet digital PCR (ddPCR) has emerged as a superior method for ctDNA detection because of its high analytical sensitivity [17,18,19,20]. The ddPCR technique involves partitioning samples through limiting dilutions, resulting in most reactions containing zero to one molecule [21]. This reduces reaction complexity, enabling more precise and reliable measurement of low mutant fractions. Unlike real-time PCR, ddPCR offers absolute quantification of target sequences [22] and improved precision and reproducibility [23].

In this study, we aimed to evaluate the analytical and clinical performance of a droplet digital PCR assay using three MSI markers, *BAT-26*, *ACVR2A*, and *DEFB105A/B*, for MSI detection in colorectal, gastric, and endometrial cancers.

## 2. Materials and Methods

### 2.1. Cell Lines and Clinical Samples

**Figure 1:**
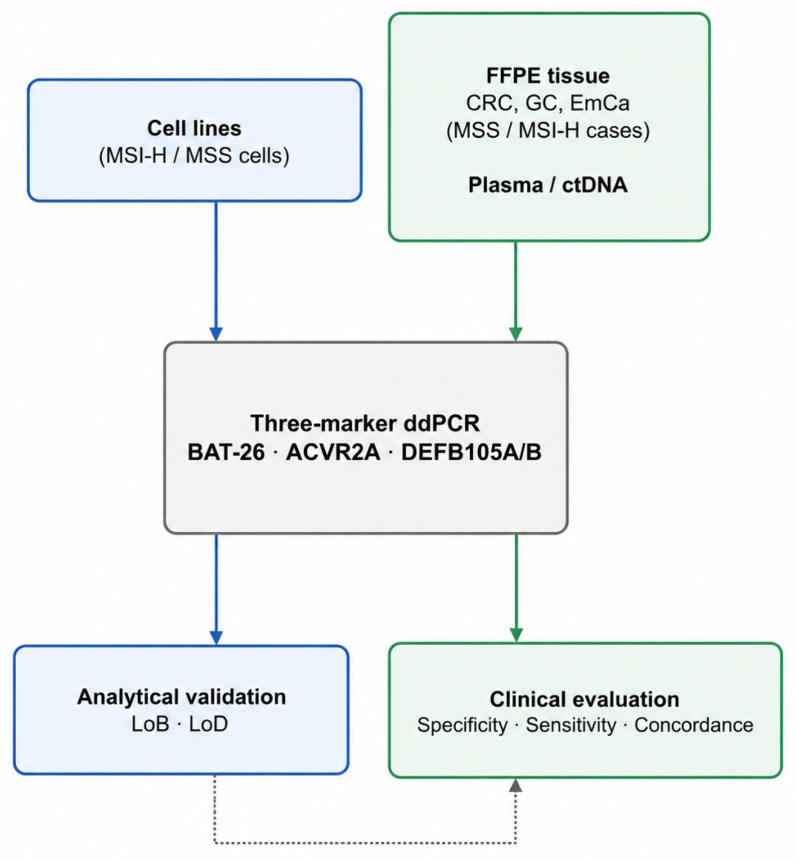
Overall study workflow, including sample types and analytical and clinical performance evaluation procedures for the three-marker droplet digital PCR (ddPCR) assay.

Human cancer cell lines SNU-1566 (colorectal cancer) and NCC-59 (gastric cancer) were used as MSI-H controls. SNU-2172 (colorectal cancer) and NCC-19 (gastric cancer) were used as MSS controls. Formalin-fixed paraffin-embedded (FFPE) samples from 83 patients with colorectal, 44 with gastric, and 63 with endometrial cancers who underwent surgical resection at Seoul National University Hospital were collected. Twenty-one plasma samples were collected from patients with metastatic solid tumors who underwent liquid biopsy at the time of diagnosis at Seoul National University Bundang Hospital. Overall study workflow is shown in Figure 1. The Seoul National University Bundang Hospital Institutional Review Board approved this study (X-2604-1041-901), and all procedures were conducted in accordance with the relevant guidelines and regulations.

### 2.2. Immunohistochemistry Analysis

IHC analysis was performed using anti-MLH1 (mouse monoclonal primary antibody, prediluted, clone M1; Ventana, Tucson, AZ, USA), anti-MSH2 (mouse monoclonal primary antibody, prediluted, clone G219-1129; Ventana, USA), anti-MSH6 (mouse monoclonal primary antibody, 1:50, clone 44; Cell Marque, Rocklin, CA, USA), and anti-PMS2 (mouse monoclonal primary antibody, prediluted, clone ERP3947; Ventana, USA). Samples were graded for the absence or presence of nuclear staining of MMR proteins in tumor cells compared to internal positive controls, such as non-neoplastic epithelial cells, stromal cells, and lymphocytes, in the vicinity of the tumor. Each case was classified into one of three categories according to the intensity and proportion of tumor nuclear staining in the whole slide as follows: (1) “intact expression” was defined as unequivocal nuclear staining in all tumor cells with clear staining of internal control tissue adjacent to the tumor cells, (2) “loss of expression” was defined as unequivocal loss of nuclear staining in all tumor cells with clear staining of internal control tissue in the vicinity of the tumor, and (3) “focal loss of expression” was defined as clearly demarcated regional loss of tumor nuclear staining with internal control tissue adjacent to the tumor cells showing clear staining.

### 2.3. Microsatellite Instability Analysis Using Pentaplex Panel

DNA was extracted from macro-dissected FFPE tumor tissue slides and corresponding normal FFPE samples using a Maxwell RSC FFPE Plus DNA Kit (Promega, Madison, WI, USA). DNA concentration and purity were evaluated with a NanoDrop spectrophotometer (Thermo Scientific, Wilmington, DE, USA), and samples with a minimal concentration of 15 ng/µL were used for analysis. MSI status was evaluated using a pentaplex panel comprising five mononucleotide repeat markers (*BAT-25*, *BAT-26*, *NR-21*, *NR-24*, and *NR-27*). PCR amplification was performed in a final reaction volume of 25 μL containing 30 ng of genomic DNA, fluorescently labeled primer sets (10 pmol/μL each), and 2× Platinum II Hot Start PCR Master Mix (Invitrogen, Waltham, MA, USA). Reactions were carried out on a SimpliAmp thermal cycler (Applied Biosystems, Foster City, CA, USA) under the following cycling conditions: initial denaturation at 95 °C for 3 min, followed by 40 cycles of 94 °C for 15 s, 58 °C for 15 s, and 72 °C for 30 s, with a final extension at 72 °C for 5 min. PCR products were analyzed by automated capillary electrophoresis using a SeqStudio Genetic Analyzer (Applied Biosystems, CA, USA), and fragment size determination was performed with GeneMapper Software 6 in microsatellite analysis mode (Applied Biosystems, USA). MSI status was determined by comparing allele sizes between matched tumor and normal samples. MSI was considered unstable if there was a shift of 2 bp or more in the tumor allele. We adopted a threshold of a size shift ≥ 2 bp, as very subtle size shifts of one base pair were observed in the pMMR cases and were considered possibly due to polymerase slippage. In addition, this subtle shift may result in inter-observer variability, reducing the reliability of MSI testing. Samples were classified as MSI-H when two or more markers were unstable, MSI-L when one marker was unstable, and MSS when there were no unstable markers. The results of PCR assay with pentaplex panel were used as the molecular reference for MSI classification.

### 2.4. Microsatellite Instability Analysis Using Droplet Digital PCR

For FFPE samples, DNA was extracted as described above. For plasma samples, cfDNA was isolated from 2 to 4 mL of plasma using a cobas cfDNA Sample Preparation Kit (Roche Molecular Systems, Pleasanton, CA, USA). DNA concentration was quantified using a Qubit dsDNA HS Assay Kit (Thermo Fisher Scientific, Waltham, MA, USA). Primers and probes were designed as described in a previous study (Supplementary Table S1) [24]. ddPCR assays were performed using the Bio-Rad QX200 system with reaction mixtures containing 900 nM of each primer and 250 nM of each probe. Thermal cycling conditions consisted of an initial enzyme activation step at 95 °C for 10 min, followed by 40 amplification cycles at 94 °C for 1 min and either 60 °C for 1 min 30 s (for *BAT-26* and *DEFB105A/B*) or 57 °C for 1 min 30 s (for *ACVR2A*), followed by a final incubation at 98 °C for 10 min. Cluster thresholding and quantification were performed with QuantaSoft v.1.7.4 (Bio-Rad). Representative plots showing droplet cluster separation and mutant quantification using this method are provided in Supplementary Figure S1. Samples were classified as MSI-H when two or more markers out of three were unstable, MSI-L when one marker was unstable, and MSS when there were no unstable markers.

### 2.5. Statistical Analysis

Statistical analyses were performed using McNemar’s test to compare differences in sensitivity and specificity between the markers for each cancer type. Using the Clopper–Pearson method, 95% confidence intervals (CI) were computed. Receiver operating characteristic (ROC) analysis was performed to evaluate each ddPCR marker as a single continuous indicator for discriminating MSI-H cases determined by reference pentaplex PCR assay. No separate training–validation split was performed. For each marker, the area under the ROC curve (AUC) was calculated and reported with a 95% CI using DeLong’s method. Optimal cut-off values were selected by maximizing Youden’s index. Sensitivity and specificity at each selected cut-off were calculated, and their 95% CIs were estimated using exact binomial CIs. All statistical analyses were performed using R 4.2.1 (R Foundation for Statistical Computing, Vienna, Austria), and *p* values less than 0.05 were considered significant.

## 3. Results

### 3.1. Limit of Blank (LoB) and Limit of Detection (LoD) of ddPCR Assay

The LoB was determined as follows: LoB = false-positive mutant allele frequency (MAF) mean + 95% confidence interval (CI). The false-positive mean and associated 95% CI were calculated through the analysis of 20 replicates of MSS cell line gDNA (NCC-19 for *BAT-26*; SNU-2172 for *ACVR2A* and *DEFB105A/B*). The LoB of *BAT-26*, *ACVR2A*, and *DEFB105A/B* were 0.095%, 0.061%, and 0.0%.

**Figure 2:**
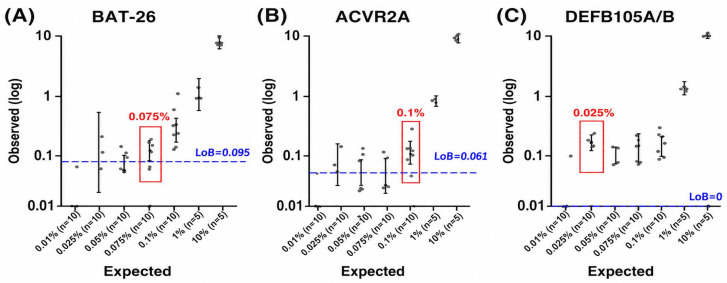
The limit of blank (LoB) and limit of detection (LoD) of droplet digital PCR for detection of microsatellite instability (MSI) with (**A**) *BAT-26*, (**B**) *ACVR2A*, and (**C**) *DEFB105A/B*.

For LoD, multiple serial dilutions of MSI cell line gDNA (NCC-59 for *BAT-26*; SNU-1566 for *ACVR2A* and *DEFB105A/B*) in MSS cell line gDNA (1%, 0.1%, 0.075%, 0.050%, 0.025%, 0.01%) were tested in 10 replicates. We considered MAF values statistically significant when replicates presented average values and standard deviation (SD) above the LoB, identifying an LoD of 0.075% for *BAT-26*, 0.1% for *ACVR2A*, and 0.025% for *DEFB105A/B* (Figure 2).

### 3.2. Clinical Cut-Off Value of ddPCR Assay

**Figure 3:**
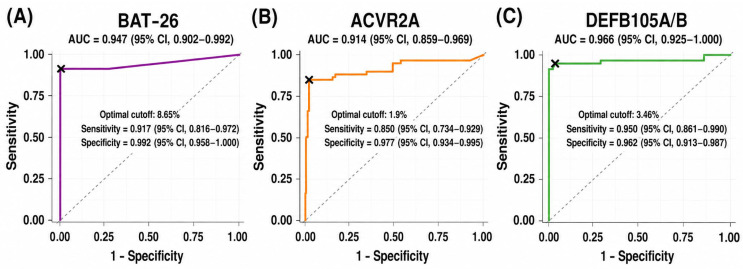
Receiver operating characteristic (ROC) curve analysis showing the area under the curve (AUC) for determining the optimal threshold of droplet digital PCR (ddPCR) for prediction of microsatellite instability (MSI) for (**A**) *BAT-26*, (**B**) *ACVR2A*, and (**C**) *DEFB105A/B*. A total of 190 samples were included in the ROC analysis, comprising 60 MSI-H and 130 MSS cases as determined by the pentaplex PCR assay.

As applying the LoD of the ddPCR assay as the cut-off for MSI determination in tissue samples resulted in a high false-positive rate, we established a clinical cut-off using the AUC to determine the optimal threshold for MSI prediction (Figure 3). The reference criteria were the results of conventional PCR assay with pentaplex panel, which are currently considered gold standard in molecular analysis [25,26].

### 3.3. Comparison of the Results Between IHC Analysis, PCR Assay with Pentaplex, and ddPCR Assay

The results of IHC analysis, PCR assay with pentaplex, and ddPCR assay are compared in Table 1 for colorectal, gastric, and endometrial cancers.

**Figure 4:**
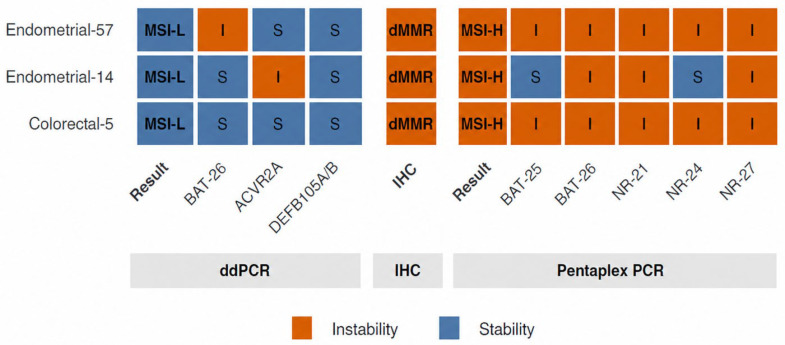
Heatmap showing discordant cases between the ddPCR assay and the reference assays for microsatellite instability (MSI) classification. Results of ddPCR assay were compared with immunohistochemistry (IHC) and pentaplex PCR assay results in individual cases.

The concordance rate between pentaplex PCR assay and the ddPCR assay was 98.4% [95% CI, 95.5–99.7]. There were three discordant cases in classification of MSI status between pentaplex PCR assay and the ddPCR assay (Figure 4).

### 3.4. Sensitivity and Specificity of Individual Markers in ddPCR Assay

The sensitivity and specificity for the detection of MSI for each marker in ddPCR assay were evaluated based on the results of conventional PCR analysis with pentaplex panel (Table 2). The sensitivity of each marker was calculated as the percentage of cases in which the marker showed instability among the cases classified as MSI-H by MSI analysis. Specificity was calculated as the percentage of cases in which the marker showed stability among the cases classified as MSS/MSI-L by MSI analysis. For colorectal cancer cases, all three markers, *BAT-26*, *ACVR2A*, and *DEFB105A/B*, missed only one MSI-H case exhibiting 96.9% sensitivity. For gastric cancer, *BAT-26* and *DEFB105A/B* showed instability in all MSI-H cases, whereas *ACVR2A* showed a lower sensitivity of 75.0% (*p* = 0.046). For endometrial cancer, *DEFB105A/B* showed a higher sensitivity (83.3%) than *BAT-26* (66.7%) and *ACVR2A* (66.7%) with no statistically significant differences. In terms of specificity, *BAT-26* and *DEFB105A/B* showed 100% specificity and *ACVR2A* exhibited 96.1% specificity in colorectal cancer. In gastric cancer, *BAT-26* showed 100% specificity, followed by *ACVR2A* (92.9%) and *DEFB105A/B* (85.7%, *p* = 0.046). In endometrial cancer, *BAT-26* and *ACVR2A* showed 100% specificity and *DEFB105A/B* exhibited 98.0%. There was no statistically significant differences in specificity between the markers for colon cancer and endometrial cancer.

### 3.5. Determination of MSI in Plasma Samples Using ddPCR Assay

**Figure 5:**
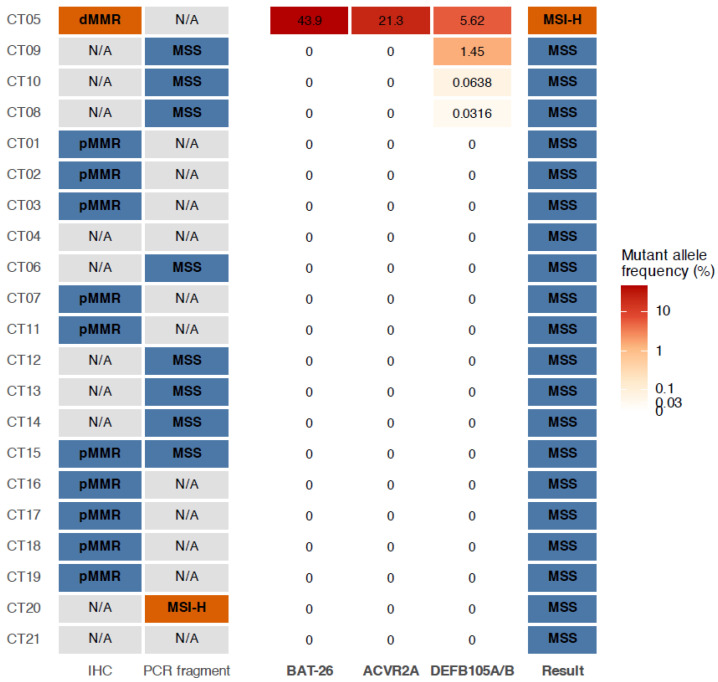
Comparison of MSI status between tissue-based conventional assays and plasma ddPCR assay in individual cases. The left panel shows the results of conventional tissue-based assays, including PCR fragment analysis and immunohistochemistry (IHC) staining. The middle panel displays the mutant allele frequencies (MAFs) of the three plasma ddPCR markers (BAT-26, ACVR2A, and DEFB105A/B) for each case. The right panel indicates the final plasma ddPCR classification. Cases are ordered according to plasma ddPCR result and maximum MAF. MSS, microsatellite stable; MMR, mismatch repair; pMMR, proficient mismatch repair; dMMR, deficient mismatch repair; N/A, not available.

A total of 21 plasma samples from patients with metastatic solid tumors (13 colorectal cancers, 6 gastric cancers, 1 hepatocellular carcinoma, and 1 pancreatic cancer) were analyzed using the ddPCR assay (Figure 5). Among these, 19 cases had available matched tissue results based on PCR fragment analysis and/or IHC staining; 17 cases were classified as MSS and/or pMMR, and 2 cases were classified as MSI-H (1 pancreatic cancer) or dMMR (1 colorectal cancer).

Using the ddPCR assay, MSI-H was detected in 1 sample (4.8%), while the remaining 20 samples (95.2%) were classified as MSS. The MSI-H result was observed in a colorectal cancer case that was also classified as dMMR by IHC analysis of the corresponding tissue sample. In contrast, the pancreatic cancer case, which was classified as MSI-H by PCR fragment analysis of the corresponding tissue sample, was classified as MSS by the ddPCR assay using plasma sample.

Among the 19 cases with available matched tissue results, 18 plasma samples showed concordant results with tissue-based MSI classification, yielding a concordance rate of 94.7% [95% CI, 74.0–99.9].

## 4. Discussion

This study evaluated the performance of a ddPCR assay using three MSI markers, *BAT-26*, *ACVR2A*, and *DEFB105A/B*, for MSI detection in colorectal, gastric, and endometrial cancers. We selected the three markers of ddPCR assay based on a previous report describing ddPCR strategy for detection of microsatellite instability [24]. *BAT-26* is one of the representative well-known markers, and *ACVR2A* and *DEFB105A/B* are novel discriminatory microsatellite markers found in previous studies [8,27].

The ddPCR assay showed high analytical sensitivity, with a LoD of 0.1% or lower, which allows for its use as a promising tool to detect MSI in liquid biopsies. These results were not significantly different from those found by Silveira et al., in which the LoD of *BAT-26* was 0.04% and that of *ACVR2A* and *DEFB105A/B* was 0.08%.

However, when applied to solid biopsies, using these LoD cut-offs for determining MSI status was not practical, as most MSS samples were misclassified as MSI. This indicates that the LoD values established with cell line samples were too sensitive for solid tissue samples. Although further studies with larger sample sizes are needed, it appears that individual cut-offs should be established for each sample type when using ddPCR assays for optimal determination of MSI status.

When clinical cut-offs determined by receiver operating characteristic analyses were applied for solid biopsies, the concordance in MSI status classification between the ddPCR assay and the PCR assay with the pentaplex panel was very high (98.4%; 95% CI, 95.5–99.7%). Among the three discordant cases, one was dMMR colorectal cancer, which was classified as MSI-H by PCR with pentaplex panel. However, no marker in the ddPCR assay showed instability. The other two discordant cases were dMMR ones in endometrial cancer. The PCR assay with pentaplex panel classified them as MSI-H, while ddPCR assay classified them as MSI-L because only one marker (*BAT-26* or *ACVR2A*) showed instability in each case. The relatively lower sensitivity observed in endometrial cancer likely reflects tumor-specific differences in microsatellite instability patterns, indicating that the selected markers may not adequately capture MSI-associated alterations in this tumor type. Previous studies have demonstrated that the frequency and distribution of unstable microsatellite loci vary substantially across tumor types, and certain loci may exhibit lower instability rates in endometrial cancer compared with colorectal cancer [8,27]. In addition, intratumor heterogeneity may contribute to discordant results by generating subclonal MSI alterations that are not uniformly represented across tumor regions or DNA aliquots [28]. Technical factors may also have influenced marker performance, including differences in amplification efficiency, fragment length variability, and low mutant allele fractions near the clinical cut-off thresholds. These findings suggest that a universal marker panel may not provide equivalent diagnostic performance across all tumor types, and they support the need for tumor-specific optimization or incorporation of additional markers to improve MSI detection in endometrial cancer.

At the individual marker level, the high-sensitivity rankings varied by cancer type. For colorectal cancer, all markers showed high sensitivity, missing only 1 case out of 32 (96.9%; 83.8–99.9%). For gastric cancer, *BAT-26* and *DEFB105A/B* showed 100% (79.4–100%) sensitivity, while *ACVR2A* showed a lower sensitivity of 75.0% (47.6–92.7%). The lower sensitivity of *ACVR2A* in gastric cancer may reflect differences in the prevalence or stability of *ACVR2A*-associated frameshift alterations compared with colorectal cancer. In addition, variability in tumor purity or regional heterogeneity may also reduce the detectability of unstable alleles in certain gastric cancer samples. For endometrial cancer, all the markers showed relatively low sensitivity; *DEFB105A/B* showed relatively higher sensitivity (83.3%; 51.6–97.9%) compared to the other markers, which exhibited a lower sensitivity of 66.7% (34.9–90.1%). The ddPCR assay with these three markers showed suboptimal performance with MSI-H endometrial cancer, underscoring the need to develop optimal panels for each cancer type. Most clinical laboratories use the same marker panel for MSI testing across all tumor types. However, because marker performance varies between tumor types, tumor-specific marker selection and panel configuration strategies should be considered. Incorporation of additional highly informative loci or optimization based on tumor-specific MSI profiles may further improve the diagnostic performance of ddPCR-based MSI assays, particularly in tumor types such as endometrial and gastric cancers.

In the analysis of plasma samples, the ddPCR assay demonstrated the feasibility of detecting MSI status using ctDNA. Among the 19 plasma samples with available matched tissue results, 1 case showed discordance with the corresponding tissue result. This case was classified as MSS by the ddPCR assay, whereas the corresponding tissue result was MSI-H. This discrepancy may reflect the limited sensitivity of ctDNA-based detection, particularly in tumors with low circulating tumor DNA fractions. The relatively low detection rate of MSI-H in plasma samples may be attributed to the limited fraction of ctDNA, especially in patients with low tumor burden or early-stage disease [29,30]. In addition, biological factors such as tumor shedding and intratumoral heterogeneity may further influence the detectability of MSI in plasma [31,32,33]. This limitation is particularly relevant in tumor types such as pancreatic cancer, which are known to exhibit low ctDNA shedding, potentially leading to false-negative results despite MSI-H status in tissue [34]. Furthermore, when applying the analytical cut-off for MAF, three false-positive cases were observed for *DEFB105A/B*. Therefore, further accumulation of plasma sample data is required to determine the necessity of establishing a clinically relevant cut-off, similar to that defined for tissue samples.

The limitations of this study are that the number of cases used in the evaluation was not sufficiently large for each cancer type. In particular, the plasma cohort was limited in size, restricting a comprehensive evaluation of the clinical performance of the ddPCR assay for ctDNA-based MSI detection. In addition, this study was retrospective in nature, and comprehensive clinical information associated with ctDNA shedding and detectability, such as tumor stage, tumor burden, tumor content, treatment status, and ctDNA fraction, was not consistently available. These factors may substantially influence plasma ddPCR performance and the interpretation of discordant or false-negative results. Further prospective studies with larger sample sizes and comprehensive clinical information are needed to clarify the operability of ddPCR-based ctDNA analysis in the clinical setting and to establish the optimal marker configuration for MSI determination.

MSI detection with ctDNA using conventional PCR assays is difficult due to its relatively low sensitivity. Therefore, the ddPCR assay can be a promising tool for ctDNA, offering high sensitivity and quantitative capabilities. This study is meaningful in that it demonstrated the feasibility of ddPCR assay for determination MSI status. As ddPCR assay is a highly sensitive and quantitative method, it could be an optimal liquid biopsy tool to determine MSI status and for longitudinal monitoring of MSI status and treatment response. This study highlights the necessity of sample-type-specific clinical cut-offs and tumor-specific marker optimization for ctDNA-based MSI detection. Further prospective studies with a large number of plasma samples will elucidate the real world applicability of ddPCR assay for detection and monitoring of MSI using ctDNA.

## 5. Conclusions

We evaluated the performance of a ddPCR assay using three markers, *BAT-26*, *ACVR2A*, and *DEFB105A/B*, in MSI detection. Determining tumor MSI status is critical for the diagnosis of Lynch syndrome and for determining treatment options for patients with various solid tumors. The ddPCR assay showed a high concordance rate with the conventional PCR assay using a pentaplex panel, which is considered the gold standard in MSI classification. We also demonstrated the feasibility of detecting MSI status using ctDNA by ddPCR assay with 21 plasma samples. Further studies are necessary to validate these markers using a greater number of plasma samples and to better understand the clinical utility of ddPCR for MSI detection and monitoring using ctDNA.

## Figures and Tables

**Table 1:** Comparison of the results by IHC analysis, pentaplex PCR assay, and ddPCR assay in tissue samples from colorectal, gastric, and endometrial cancers.

Cancer Types	IHC Analysis	Pentaplex PCR Assay			ddPCR Assay		
		MSS	MSI-L	MSI-H	MSS	MSI-L	MSI-H
Colorectal cancer	dMMR	0	0	32	1	0	31
	pMMR	50	1	0	49	2	0
Gastric cancer *	dMMR	0	0	14	0	0	14
	fMMR	0	0	1	0	0	1
	pMMR	24	0	0	21	3	0
Endometrial cancer	dMMR	1	0	11	0	3	9
	fMMR	0	0	1	0	0	1
	pMMR	49	1	0	50	0	0

Abbreviations: ddPCR, droplet digital PCR; IHC, immunohistochemistry; MSI, microsatellite instability. * IHC analysis was not performed for five cases of gastric cancer; the pentaplex PCR assay and ddPCR assay showed concordant results in all cases (three MSS and two MSI-H).

**Table 2:** Sensitivity and specificity of individual markers in droplet digital PCR (ddPCR) assay using tissue samples from colorectal, gastric, and endometrial cancers.

Marker	Colorectal Cancer		Gastric Cancer		Endometrial Cancer	
	Sensitivity [95% CI]	Specificity [95% CI]	Sensitivity [95% CI]	Specificity [95% CI]	Sensitivity [95% CI]	Specificity [95% CI]
*BAT-26*	96.9 (31/32) [83.8–99.9]	100 (51/51) [93.0–100]	100 (16/16) [79.4–100]	100 (28/28) [87.7–100]	66.7 (8/12) [34.9–90.1]	100 (51/51) [93.0–100]
*ACVR2A*	96.9 (31/32) [83.8–99.9]	96.1 (49/51) [86.5–99.5]	75.0 (12/16) [47.6–92.7]	92.9 (26/28) [76.5–99.1]	66.7 (8/12) [34.9–90.1]	100 (51/51) [93.0–100]
*DEFB105A/B*	96.9 (31/32) [83.8–99.9]	100 (51/51) [93.0–100]	100 (16/16) [79.4–100]	85.7 (24/28) [67.3–96.0]	83.3 (10/12) [51.6–97.9]	98.0 (50/51) [89.6–100]

## Data Availability

The datasets used and/or analyzed during the current study are available from the corresponding author on reasonable request.

## Supplementary Materials

The following supporting information can be downloaded at: https://www.mdpi.com/article/10.3390/diagnostics16101550/s1, Figure S1: Representative droplet digital PCR (ddPCR) scatterplots showing cluster separation and mutant allele quantification; Table S1: Primers and probes for each marker.
