# Supplementary material for: Evaluation of Droplet Digital PCR Assay for the Detection of Microsatellite Instability in Colorectal, Gastric, and Endometrial Cancers
Source: Diagnostics (Basel). 2026 May 20;16(10):1550. doi: 10.3390/diagnostics16101550 (PMC13205154; doi:10.3390/diagnostics16101550)

Supplementary Figure 1. Representative droplet digital PCR (ddPCR) scatterplots showing cluster separation and mutant allele quantification. The upper panels represent clinical samples with high mutant allele frequency (MAF), whereas the lower panels represent samples with low MAF.

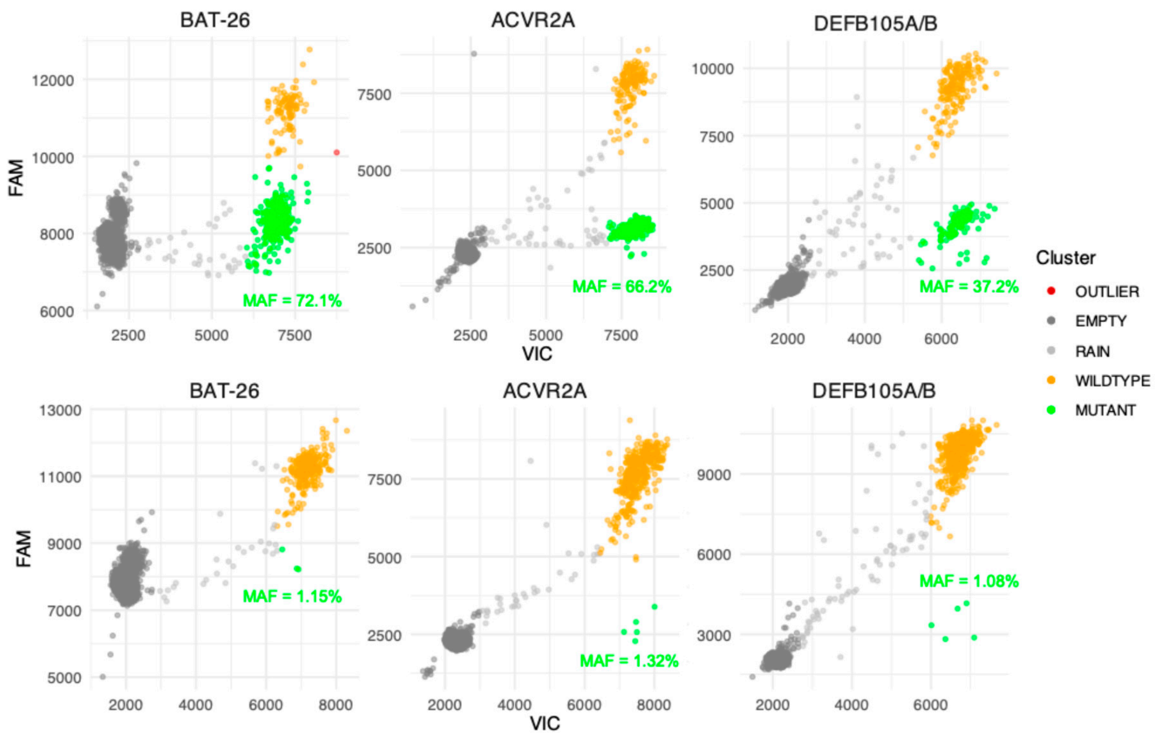

Supplement: Supplementary file 1 [file diagnostics-16-01550-s001.zip › diagnostics-4318349-Supplementary Figure S1.pdf]
