# Supplementary material for: Evaluation of Droplet Digital PCR Assay for the Detection of Microsatellite Instability in Colorectal, Gastric, and Endometrial Cancers
Source: Diagnostics (Basel). 2026 May 20;16(10):1550. doi: 10.3390/diagnostics16101550 (PMC13205154; doi:10.3390/diagnostics16101550)
Supplement: Supplementary file 1 [file diagnostics-16-01550-s001.zip › diagnostics-4318349-Supplementary Table S1.pdf]

Supplemental Table 1. Primers and probes for each marker

Marker	Forward primer	Reverse primer	Probe 1	Probe 2
BAT-26	gacttcagccagtatatgaaattggatattg	gtatatgtcaatgaaaacatttttaaccattcaac	aggtaaaaaaaaaaaaaaaaaaaaaaaaaagg	agcagtcagagcccttaaccttt
ACVR2A	gaggaggaaattggccagcatc	agctaactggataacttacagcatg	cctcttttttttatgc	acttcctgcatgtcttcaagag
DEFB105A/B	ttgaaaaatctgggctgattcttga	tgagggagctttccaggaaatg	tcccttttttttttggt	ctttgacatgttccccatttctag
